# From Almost Empty to Half Full? A Response to Recent Commentaries

**DOI:** 10.15171/ijhpm.2016.94

**Published:** 2016-07-17

**Authors:** Lisa Forman, Gorik Ooms, Claire E. Brolan

**Affiliations:** ^1^Dalla Lana School of Public Health, University of Toronto, Toronto, ON, Canada.; ^2^Institute of Public Health, Heidelberg University Hospital, Heidelberg, Germany.; ^3^School of Public Health, University of Queensland, Brisbane, QLD, Australia.

In his 2005 book, *Death with Interruptions,* which imagines a world in which people stop dying*,* Jose Saramago tartly describes the ethical debates that ensue in the following way:

“The philosophers, divided as always between frowning pessimists and smiling optimists, readied themselves to recommence for the thousandth time the ancient dispute over whether the glass was half full or half empty.”^1^ Discussions about the right to health and global health policy are unfortunately poised around the diametrically opposed social justice dilemma of appropriate policy responses in a world in which people die all too easily from avoidable and foreseeable causes. Yet debates around the right to health often approximate these kinds of half full-half empty disputes: yes we have made tremendous gains in the last 30 years, yes we have tremendously far to go. We are grateful that three thoughtful responses to our paper eschewed these kinds of zero-sum approaches and instead sketched out important context and gaps in our analysis of the Sustainable Development Goal (SDG) agenda in “Rights Language in the Sustainable Development Agenda.”^2^

We wrote that paper in order to assess the extent to which right to health advocacy has influenced the health goals in major reports in the SDG process. To some extent this inquiry reflected several years of our professional work as part of the Go4Health Consortium, formed precisely to advance rights-based approaches to the SDG’s health goals. We acknowledge that our optimism may reflect wishful thinking about the extent of gains achieved! The comments on our paper written by Rushton,^3^ Hawkes and Buse,^4^ and Williams and Blaiklock,^5^ insightfully identify and analyse the gaps and limitations of this analysis and of rights discourse in the SDGs. In this response, we identify four primary insights from these papers that effectively illuminate these gaps as well as outline future directions for research on human rights and global health policy. We believe that these commentaries considerably enrich the debate about what human rights and especially the right to health may offer to global health policy.

*First*, as all the authors point out, without effective monitoring and accountability, the aspirations of the SDGs are at risk of remaining simply that. Hawks and Buse put this plainly: “What the SDG framework currently, and crucially lacks, is a serious and explicit commitment to accountability for the goals, targets and even the underlying principles (such as the human rights principle) …. [I]f we are to get serious about the realization of the right to health within the SDGs, we need to move beyond the rhetoric of goals and targets and establish realistic, feasible, and responsible plans for action.”^4^ We are entirely in agreement, and view human rights norms, laws and tools as offering particularly important mechanisms for assuring accountability. Litigation and rights-based advocacy in particular will assist civil society globally in holding governments to account for their compliance with SDG commitments.

*Second*, Williams and Blaiklock indicate that “discourse analysis needs to extend, to examine where and how human rights are used, and as importantly, where they are not used.”^5^ In particular they observe “the phrasing around the rights-related terms avoids recognition of the obligations of States and non-state duty bearers, and fails to address rights as (legal entitlements).”^5^ Williams and Blaiklock argue that the danger of this kind of unlinked rights language is particularly acute in the context of the neoliberal emphasis of global health policy which privileges private sector and civil and political rights. They suggest that if discourse in global health policies does not go beyond its current usage, “then these agreements could encourage superficial approaches to human rights in future agreements and policies.”^5^ Again, we are entirely in agreement, and believe that this analysis points very clearly and usefully towards what right to health advocates should be arguing for in future global health policy initiatives.

*Third*, regarding our contention that universal health coverage **(**UHC) correlates with the right to health, Rushton rightly points out that “if UHC equates with a right, it is with the right to access healthcare services—an important aim in itself, but one which is only a part of the wider right to health…[which] must implicate not only health services but also the social and economic determinants of health status.”^3^ At the risk of becoming repetitive, we agree entirely, indeed this is an elision we have explored in previous scholarship.^6^

*Finally*, Rushton points out that the inclusion of sexual and reproductive health rights (SRHR) landed up being stronger than the right to health in the final SDGs. We believe that this is a particularly important outcome for right to health advocates to mull over: while the right to health is a contested human right, it is not more so than sexual and reproductive health rights which arguably rest upon far more challenging ideological ground. Yet SRHR advocates were able to effectively transcend trigger points that would exclude these interests and were able to achieve important explicit protections in the final SDGs. How SRHR advocates achieved this outcome should occupy the thinking of right to health advocates as we gear up for future global health policy exercises.

## Ethical issues

Not applicable.

## Competing interests

Authors declare that they have no competing interests.

## Authors’ contributions

LF drafted the response, GO and CB commented and revised.

## Authors’ affiliations

^1^Dalla Lana School of Public Health, University of Toronto, Toronto, ON, Canada. ^2^Institute of Public Health, Heidelberg University Hospital, Heidelberg, Germany. ^3^School of Public Health, University of Queensland, Brisbane, QLD, Australia.
